# Entire genome sequence analysis of genotype IX Newcastle disease viruses reveals their early-genotype phylogenetic position and recent-genotype genome size

**DOI:** 10.1186/1743-422X-8-117

**Published:** 2011-03-14

**Authors:** Xusheng Qiu, Qing Sun, Shuang Wu, Li Dong, Shunling Hu, Chunchun Meng, Yantao Wu, Xiufan Liu

**Affiliations:** 1 key Laboratory of Animal Infectious Diseases, Yangzhou University, Yangzhou 225009, PR China; 2 Shanghai Veterinary Institute, Chinese Academy of Agricultural Sciences, Shanghai 200241, PR China

## Abstract

**Background:**

Six nucleotide (nt) insertion in the 5'-noncoding region (NCR) of the nucleoprotein (NP) gene of Newcaslte disease virus (NDV) is considered to be a genetic marker for recent genotypes of NDV, which emerged after 1960. However, F48-like NDVs from China, identified a 6-nt insert in the NP gene, have been previously classified into genotype III or genotype IX.

**Results:**

In order to clarify their phylogenetic position and explore the origin of NDVs with the 6-nt insert and its significance in NDV evolution, we determined the entire genome sequences of five F48-like viruses isolated in China between 1946 and 2002 by RT-PCR amplification of overlapping fragments of full-length genome and rapid amplification of cDNA ends. All the five NDV isolates shared the same genome size of 15,192-nt with the recent genotype V-VIII viruses whereas they had the highest homology with early genotype III and IV isolates.

**Conclusions:**

The unique characteristic of the genome size and phylogenetic position of F48-like viruses warrants placing them in a separate geno-group, genotype IX. Results in this study also suggest that genotype IX viruses most likely originate from a genotype III virus by insertion of a 6-nt motif in the 5'-NCR of the NP gene which had occurred as early as in 1940 s, and might be the common origin of genotype V-VIII viruses.

## Background

Newcastle disease (ND) is one of the most serious infectious diseases of birds causing major economic losses in poultry industry[1-3]. Its causative agent, virulent Newcastle disease virus (NDV), belongs to the genus *Avulavirus*, in the subfamily *Paramyxovirinae*, family *Paramyxoviridae*, order *Mononegaviriales*[4,5]. NDVs have a negative-sense, single-stranded continuous RNA genome about 15,186-nt, 15,192-nt or 15,198-nt in length [6-8] that contains six genes in the order of 3'-NP-P-M-F-HN-L-5', encoding six viral proteins (nucleoprotein, phosphoprotein, matrix protein, fusion protein, haemagglutinin-neuraminidase and large protein, respectively)[9]. Two additional proteins, V and W, are expressed by mRNAs derived from the P gene via RNA editing [10,11].

Phylogenetically, NDVs have been classified into two major divisions, class I and class II [8,12]. Class I NDVs with the genome size of 15,198-nt are occasionally isolated from wild aquatic birds and domestic poultry and all but one of them are avirulent [8,13-16]. Class II viruses include most virulent and some avirulent NDVs: genotypes I-IV viruses are early lineage before 1960 with the genome size of 15,186-nt; whereas genotypes V-VIII are recent lineage after 1960 with the genome size of 15,192-nt [4,7,8,17,18]. Genotype I of class II contains mainly avirulent isolates from wild waterfowl and poultry species of the world; genotype II consists of North American isolates, which display different virulence ranging from lentogenic, mesogenic to velogenic; genotypes III and IV viruses represent early isolates from the Far East and Europe respectively during the first pandemic from mid 1920 s to late 1950 s; NDV strains isolated from the second pandemic during 1960 s and 1970 s belong to new genotypes V and VI; subtype VIb viruses are responsible for the third pandemic of pigeon origin during the 1980 s; novel genotypes of VIII and VII (many subgenotypes) which result in the fourth and latest pandemic have emerged since late 1980 s in the Far East, Europe, and South Africa [8,19-21].

NDV strain F48 ("F48E8" or "F48E9" was used in previous publications in which E8 or E9 means the 8^th ^or 9^th ^egg-passage of the original virus) was isolated from a diseased chicken in Northern China in 1946 and has been used as standard challenge strain for vaccine evaluation in this country [21-23]. The phylogenetic grouping of F48-like viruses is controversial in the literature: genotype IX of class II by some researchers [14-16,21,23,24] while genotype III by others for their highest homology of F gene with genotype III viruses [8,25,26]. At all events, it is evident that genotype IX is a sister clade of genotype III isolates which emerged in 1930 s. On the other hand, F48-like viruses have the 6-nt insert in the 5'-NCR of NP gene, which is considered to be a genetic marker of NDV strains emerged after 1960 [7,8]. However, the full-length genome of F48-like NDVs has not been determined. In order to clarify the phylogenetic position of F48-like viruses and explore the origin of NDVs with 6-nt insert and its significance in NDV evolution, five F48-like viruses isolated in China between 1946 and 2002 were characterized and sequenced.

## Results

### Analysis of genome size

**Figure 1:**
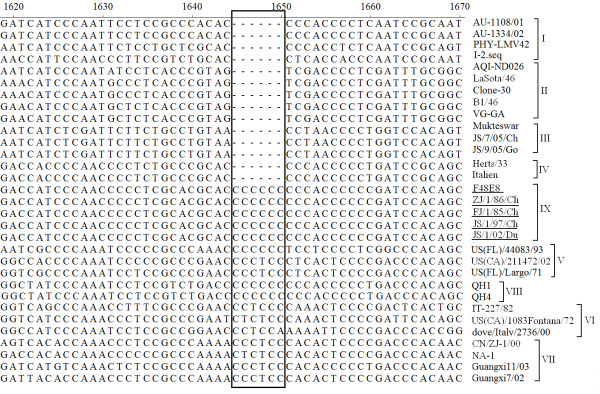
**Alignment of the 3'-terminal non-coding sequences of the NP gene in the region of 6 nt insertion**. Sequences obtained from the current study are underlined. The position of gaps were filled with '-'. The insertion site was framed.

To determine the exact genome size of F48-like NDV isolates, the full-length genome sequences were compiled from sequences of nine overlapping cDNA fragments along with the sequences of the GC-rich region of NP gene and both ends of the genomes. Those sequences were submitted to GenBank and the accession number was FJ436302 - FJ436306. The results of sequencing displayed that these F48-like NDVs carried 6-nt insert in the 5'-NCR of the NP gene, the same as that of genotypes V-VIII NDVs which emerged after 1960 s (see Figure 1). Besides, no other insert or deletion was found when compared with all known NDV isolates. Therefore, the genome size of all the five genotype IX isolates was 15,192-nt, just as predicted before. Moreover, those 5 viruses isolated during 1948-2002 shared 99% nucleotide sequence identity of their genomes and the same 6-nt insert motif CCCCCC.

### Phylogenetic analysis

**Figure 2:**
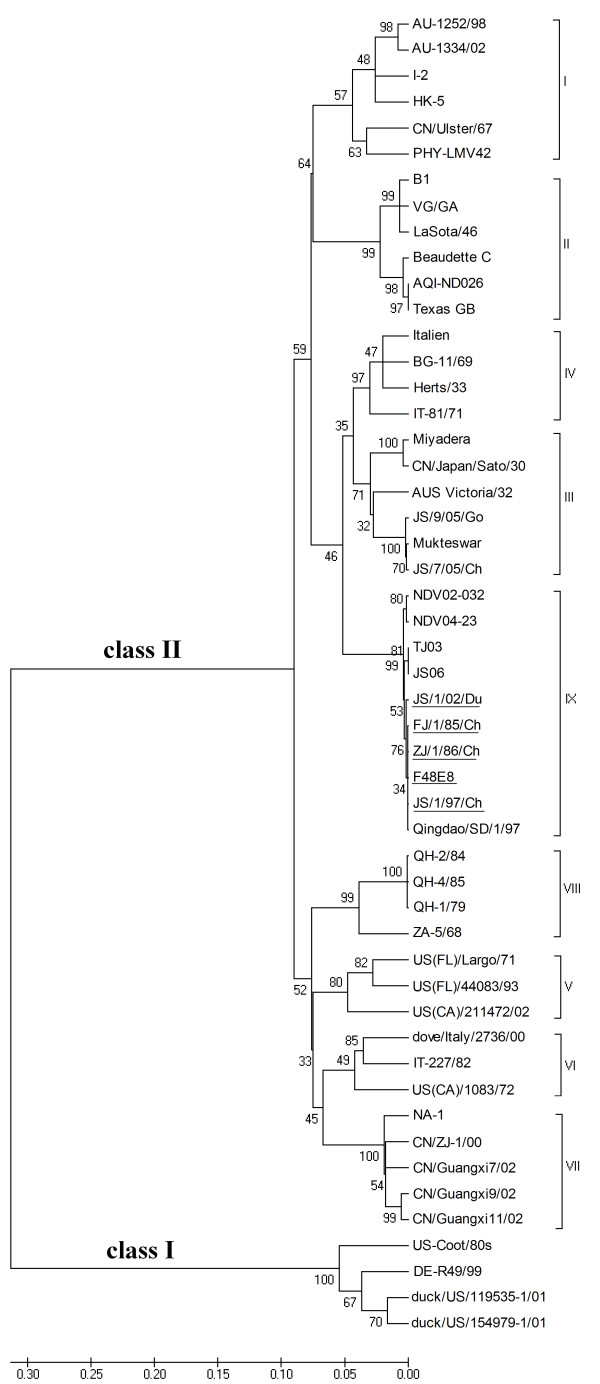
**Phylogenetic tree of NDV strains**. Tree construction was done using the Neighbor Joining method with the maximum composite likelihood substitution model for partial F gene (nt 47-420) by program MEGA version 4 (Tamura, Dudley, Nei, and Kumar 2007). Divisions and genotypes are indicated by roman numerals. Sequences obtained from the current study are underlined.

Phylogenetic analysis of the five F48-like NDV strains together with NDVs representing the established genotypes was first performed using the variable region seqences (nt 47-420) of the F gene (Figure 2). The tree consisted of two major divisions, class I and class II, the latter was further divided into two lineages, early and recent. The early lineage included five genotypes (I to IV and IX) while the recent lineage consisted of four genotypes (V to VIII). It is obvious that F48-like strains (genotype IX) were close to but diverged from the early genotypes III and IV strains, forming a separate subclade.

Table 1 shows the range of F gene sequence similarity of NDV strains within one genotype and between different genotypes. The sequence similarity of F gene between genotype IX and III was the highest, ranging from 91.2% to 94.3%. The sequence similarity of other genes between genotype IX and III NDVs was also the highest when compared with those between genotype IX and other genotypes (data not shown).

**Table 1:** Range of F gene nucleotide sequence similarity (%) of NDV strains within one genotype and between different genotypes

Genotype (No. of sequences)	I	II	III	IV	V	VI	VII	VIII	IX
**I(23)**	**>91.8**	87.0-91.8	88.6-93.3	86.2-92.5	81.9-86.3	83.4-87.9	82.6-92.2	85.8-89.7	89.3-91.7
**II(32)**	-	**>89.1**	87.1-91.2	85.1-90.0	81.2-85.3	82.3-86.4	80.9-90.1	84.8-88.0	88.5-89.9
**III(6)**	-	-	**>92.4**	87.8-94.6	83.5-88.3	83.7-90.1	83.2-89.1	87.1-91.6	91.2-94.3
**IV(5)**	-	-	-	**>92.2**	83.6-89.5	84.8-91.3	84.1-89.6	86.7-92.2	88.6-92.9
**V(11)**	-	-	-	-	**>89.2**	84.4-90.9	82.7-88.5	86.7-90.8	84.4-87.4
**VI(16)**	-	-	-	-	-	**>91.4**	84.8-92.4	88.4-92.4	84.8-88.7
**VII(45)**	-	-	-	-	-	-	**>84.6**	86.3-91.2	84.8-87.7
**VIII(2)**	-	-	-	-	-	-	-	**>93.1**	87.6-90.1
**IX(7)**	-	-	-	-	-	-	-	-	**>99.3**

The nucleotide sequence similarity of NDV strains were calculated by the MegAlign program in the Lasergene package (DNASTAR Inc. Madison, WI 53715, USA). 30 sequences of full-length genome together with more than one hundred sequences of complete F gene were involved.

**Figure 3:**
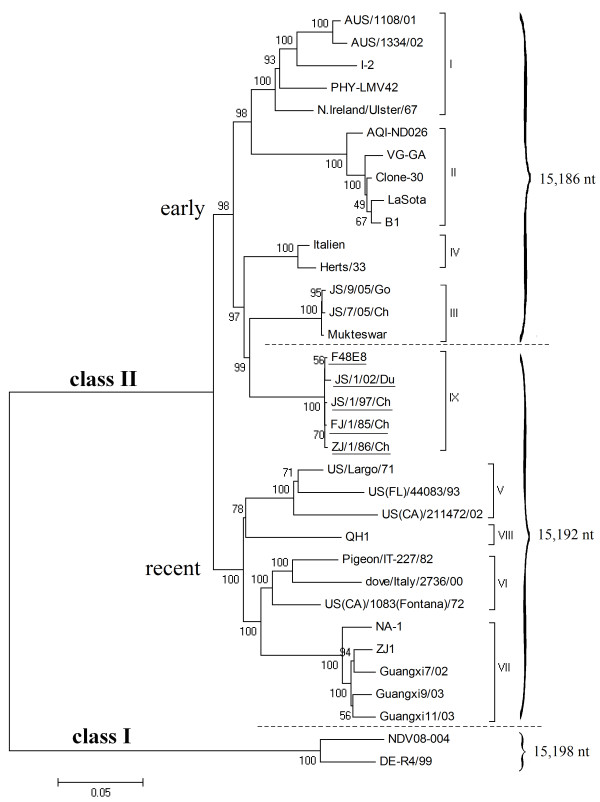
**The phylogenetic tree based on complete genome sequences showing relationship between geno-groups and genome size categories**. Tree construction was done using the Neighbor Joining method with the maximum composite likelihood substitution model by program MEGA version 4 (Tamura, Dudley, Nei, and Kumar 2007). The tree is rooted to class I sequence and all genotypes of class II NDV except genotype X are included.

Indeed, no matter which gene was used, the phylogenetic trees indicated very similar relationship of genetic groups. Figure 3 is the phylogenetic tree based on the entire genome sequences of the five F48-like NDVs in this study and those of other NDVs representing genotypes I through VIII which are available from the GenBank. The phylogenetic position of the F48-like NDVs here is consistent with the tree in Figure 2. Genotype IX strains were also clustered into early lineage, closely related with but diverged from genotypes III and IV strains.

### GC content of the genomic sequences

The GC content of the sequences of Newcastle disease virus is also an important molecular characteristic. In table 2, we calculated the GC content of different region from 25 strains of NDV, including the entire genome, 6 complete viral genes, and also the 5' NCR of NP gene in which extra 6 nt were detected. It was noted that the GC content of full-length genome of all the 25 NDV strains were similar, however, the GC content of 5' NCR of NP gene showed significant difference. The 5' NCR of NP gene of genotype IV-IX NDV strains displayed more than 60% GC content, while that of genotype I and II strains showed about 53% and 55% GC content. Interestingly, the GC content of Genotype III strains in the same region was about 58%, higher than genotype I and II NDVs but lower than genotype IV-IX NDVs.

**Table 2:** The GC contents in different regions of NDV genomes from 25 strains representing different genotypes

Genotype (No. of sequences)	Genome	NP gene	5'UTR of NP	P gene	M gene	F gene	HN gene	L gene
**I(3)**	46.56 ± 0.16	52.31 ± 0.28	**55.55 ± 0.29**	52.54 ± 0.84	49.05 ± 0.33	45.27 ± 0.09	46.91 ± 0.19	43.97 ± 0.37
**II(3)**	46.23 ± 0.05	52.25 ± 0.13	**52.90 ± 0.28**	52.58 ± 0.28	47.89 ± 0.16	44.82 ± 0.08	46.56 ± 0.03	43.77 ± 0.04
**III(3)**	46.58 ± 0.03	52.27 ± 0.00	**58.21 ± 0.50**	52.74 ± 0.07	48.59 ± 0.18	45.04 ± 0.03	47.51 ± 0.08	44.05 ± 0.02
**IV(3)**	46.68 ± 0.13	52.35 ± 0.35	**62.85 ± 0.76**	52.49 ± 0.62	49.30 ± 0.60	45.19 ± 0.13	46.32 ± 0.69	44.50 ± 0.15
**IX(3)**	46.85 ± 0.04	52.80 ± 0.03	**62.16 ± 0.28**	51.71 ± 0.13	49.48 ± 0.05	44.59 ± 0.03	48.09 ± 0.09	44.45 ± 0.06
**V(3)**	46.24 ± 0.30	52.12 ± 0.13	**64.58 ± 2.48**	52.46 ± 0.66	47.62 ± 0.17	44.95 ± 0.46	46.35 ± 0.86	44.04 ± 0.36
**VI(3)**	46.12 ± 0.34	51.66 ± 0.78	**60.39 ± 0.84**	53.04 ± 0.67	47.16 ± 0.59	45.40 ± 0.26	45.65 ± 0.33	43.80 ± 0.52
**VII(3)**	46.61 ± 0.13	51.45 ± 0.46	**62.48 ± 2.83**	53.60 ± 0.36	48.51 ± 0.20	45.12 ± 0.34	46.49 ± 0.76	44.45 ± 0.07
**VIII(1)**	46.29	51.92	**61.06**	53.64	47.32	44.84	46.29	43.94

The GC content of the full-length genome as well as the 6 viral genes was calculated by the EditSeq program in the Lasergene package (DNASTAR Inc. Madison, WI 53715, USA). Data are shown as the average of each genotype ± standard deviation

### Molecular characterization of F protein

**Figure 4:**
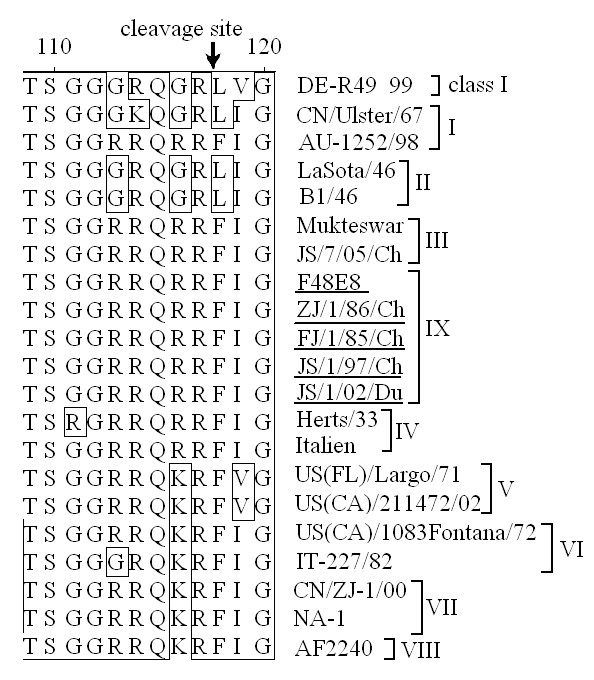
**Amino acid sequence alignment of the F protein cleavage site**. The differences in basic amino acid in the region from aa 112 to 117 are framed.

All the genotype IX strains in this study as well as other genotype IX isolates whose F gene sequences are available in the Genbank displayed the F protein cleavage site motif as 112RRQRR↓F117, the same as that of genotype III-IV strains (Figure 4). This finding is coincident with the biological characteristics of F48, which is used as the standard challenge strain in China (ICPI, 1.99). Moreover, F protein of genotype IX NDVs also had six potential N-glycosylation sites which were highly conserved among NDV isolates. The transmembrane (TM) and cytoplasmic regions of genotype IX NDVs contained several conserved substitutions and a non-conserved N for D substitution at residue 545.

### Molecular characterization of HN protein

NDV strains of different genotypes show differences in the size of the HN protein which is the major determinant for virulence. The HN protein of all genotype IX strains is 571 amino acids long, the same size as HN of genotypes III-VIII viruses. The HN proteins of genotype IX strains contained all the six sites N-linked potential glycosylation sites at position 119, 341, 433, 481, 508, and 538 [27,28]. In addition, the HN of genotype IX NDVs contained positions E401, R41 and Y526 associated with receptor binding, and residues R174, R416 and R498 involving in NA activity [29-31].

### Alignment of untranslated region of NDV genome

**Figure 5:**
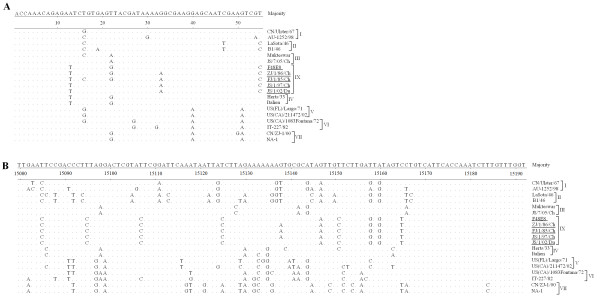
**Alignment of the leader (A) and trailer (B) sequences of genotype IX NDV with those of other genotype strains**. Genotypes are indicated. Sequences obtained from the current study are underlined. Sequences are presented as cDNA in the 5'→ 3' direction. Nucleotides that match the consensus exactly are denoted by '·'

Figure 5 shows the alignment of the leader (A) and trailer (B) sequences of genotype IX NDVs with those of other genotype strains. Genotype IX NDVs contained the same gene-start (GS) signal and gene-end (GE) signal which are highly conservative for all NDV strains. Besides, the NP-P intergenic region of only one nucleotide was G in most genotype I-II NDV strains, whereas it was A in genotype IX NDV strains as well as genotype III-VIII strains. Several unique nucleotide substitutions were found in trailer region of genotype IX viruses, for example, C15095, C15107, C15125, and C15151 (Figure 5B).

## Discussion

The outbreaks of the genotype V-VIII NDVs were still an enima in the history of NDV evolution. Where did those viruses come from? Did they evolve directly from genotype I, II or III? The genetic character that obviously differentiating those viruses from early genotypes was the six nucleotide (nt) insertion in the 5'-noncoding region (NCR) of the nucleoprotein (NP) gene.

In this study, the 5 viruses isolated from 1948 to 2002 displayed the genome size of 15,192 nt due to the same 6-nt insert CCCCCC in the NP gene. However, those NDV strains shared high identity with genotype III, and were obviously clustered in a sister clade of genotype III in phylogenetic trees (see Figure 2, 3). It is well known that genotype III is a typical "early" NDV geno-group, while 6-nt insert of NP is characteristic of "recent" genotypes[8]. That is to say, two contradictive genetic features were both identified in F48-like NDV strains, suggesting the transitional role for those viruses in NDV evolution.

It is reasonable to infer that genotype IX viruses most likely originate from an early genotype III virus by the insertion of a 6-nt motif in the 5'-NCR of the NP gene, and recent V-VIII genotypes may come from genotype IX viruses, or evolve directly from genotype III or IV viruses in the same way. There are several evidence can be provided to support this hypothesis.

Firstly, it was noteworthy that a classic genotype III strain Australia-Victoria/32 shared the highest sequence similarity with F48 strains. The nucleotide sequence identity of the F, HN and L gene of Australia Victoria/32 (AV/32) with F48 strains was 94.3%, 93.7% and 94.4% respectively. In previous studies, early lineage viruses AV/32 (genotype III) and Herts/33 (genotype IV) have been positioned as the possible progenitor of recent virulent strains according to the sequence and phylogenetic analysis based on the HN, M-F, M, P and L gene sequences respectively and also their early date of isolation [32-36]. Results in this study indicated that F48-like viruses (genotype IX) invariably shared the closest homology with AV/32 viruses and displayed the genome size of 15192-nt.

Secondly, F48 which was isolated from an ND outbreak in Northern China in 1946 [22] is the earliest NDV isolate known to have the 6-nt insert in the 5'-NCR of NP gene, which suggested that NDV strains with 15,192-nt genome size emerged as early as 1940 s, rather than 1960 s when genotype V strains came out.

The recent genotypes V-VIII strains, as early as they were first isolated, have displayed wide genetic distances and geographical distributions, which is indicative of a long period of evolution prior to the emergence of the recent viruses. On the other hand, the insertion of a 6-nt motif is an rare event in NDV evolution in view of the extremely low probability of nucleotide addition or deletion in the genome RNA of Paramyxoviruses [37,38]. Thus, it was most possible that genotype IX NDV was the common origin of genotype V-VIII.

Thirdly, it is noteworthy that all the recent viruses are virulent and their HN protein is exclusively 571 amino acids long, suggesting that their common progenitor must possess those genetic characteristics. As described below, genotype IX displayed the F protein cleavage site motif of ^112^RRQRR↓F^117 ^and a HN protein of 571 amino acids.

At last, the GC content of the full-length and partial genome of Newcastle disease virus was compared in this study (table 2). It was noted that most region of genomic sequences of all class II NDV strains shared similar GC content, however, the 5' NCR of NP gene, where the 6-nt insert was found, showed significant difference in GC content. All the viruses with 15,192-nt genome displayed more than 60% GC content, while that of most 15,186-nt genome strains were no than 50% in this region. Interestingly, F48-like viruses showed high GC content in the 5' NCR of NP gene, the same as that of recent genotypes, suggesting the relationship between genotype IX and IV-VIII.

Moreover, the significance of 6-nt insert in NP gene for NDV has never been explored. A phenomenon has been observed in this study: genotype IX viruses were isolated ranging from 1940 s to 2000 s; In comparison, the early genotype III viruses such as AV/32 from Australia, Miyadera/51 and Sato/30 from Japan and Mukteswar from India are prevalent in Australia and Asia during the first pandemic of ND before 1960 but no longer detected thereafter with the exception of Mukteswar which is used as a vaccine virus in some Asian countries[39]. Therefore, two different kinds of early genotypes NDVs, one with the genome size of 15,186-nt and the other with the genome size of 15,192 nt, exist simultaneously between 1940 s and 1960, while the former one is the predominant. In contrast, the recent genotypes V-IX viruses with genome size of 15,192-nt have emerged and predominated while early genotypes III and IV viruses have disappeared since 1960 s. It seems that NDVs with the insertion of 6-nt motif in the 5'NCR of NP gene might gain certain survive advantage in selective pressure of the ever-changing ecology.

## Conclusion

Results in this study indicated that F48-like viruses are transitional class II NDVs with early-genotype phylogenetic position and recent-genotype genome size of 15,192-nt, which makes them to be a separate geno-group, genotype IX; genotype IX viruses most likely originate from an early genotype III virus by the insertion of a 6-nt motif in the 5'-NCR of the NP gene, and recent V-VIII genotypes may come from genotype IX viruses, or evolve directly from genotype III or IV viruses in the same way; and this insertion is an important event in NDV evolution which had occurred as early as in 1940 s.

## Materials and methods

### Viruses

NDV strains for entire genome sequence analysis in this study are as follows: F48E8 (the 8^th ^egg-passaged stock of F48) was isolated from chicken outbreak in Northern China in 1946 [22]; FJ/1/85/Ch, ZJ/1/86/Ch, and JS/1/97/Ch were isolated from chickens in Eastern China in 1985, 1986 and 1997 respectively; strain JS/1/02/Du was isolated from a healthy duck in our laboratory in 2002 [21]. All the five strains have been characterized as virulent NDVs and assigned to genotype IX previously (detailed data see Table 3). They were grown in 10-day-old embryonated specific-pathogen-free (SPF) chicken eggs and the allantoic fluids were harvested and stored in -70°C until use.

**Table 3:** Background information of F48-like NDV strains used for full-length genomic sequence analysis in this study.

Strain	Original host	Year of isolation	Place of isolation	pathotype
F48E8	Chichen	1946	Beijing	VV
F/1/85/Ch	Chichen	1985	Fujian	VV
ZJ/1/86/Ch	Chichen	1986	Zhejiang	VV
JS/1/97/Ch	Chichen	1997	Jiangsu	VV
JS/1/02/Du	Duck	2002	Jiangsu	VV

"VV" stands for "very virulent".

### Preparation of viral RNA and RT-PCR

Viral genomic RNA was directly extracted from the allantoic fluid of each isolate using a Trizol RNA extraction kit (Invitrogen, Carlsbad, CA), according to the manufacturer's instructions. The cDNA was reverse transcribed from viral RNA with 6-nt random primer or a specific primer 5'-ACC AAA CAG AGA ATC-3' complementary to the 3' end of the NDV genomic RNA. A set of nine primer pairs specific for genotype III and IX isolates (see Table 4) were then used in PCR to generate successive and overlapping DNA fragments of each full-length genome from 1 μl cDNA transcript. Detailed procedure of reverse transcription and PCR was performed without modification as described elsewhere [21].

**Table 4:** Nine pairs of primers used to generate overlapping PCR fragments from genomes of F48-like NDV strains

Fragment designation		Primer sequence (5'-3')	Position	Expected product size (bp)
**A**	F^a^	ACCAAACAGAGAATCCGTGAG	1-21	2280
	R^b^	TGGACGATTTATTGCTAAGCTTG	2258-2280	
**B**	F	CAAGACTGGAGCAAGCAACT	2219-2238	2003
	R	GGAGAGGCATTTGCTATAGG	4202-4221	
**C**	F	GGGCTCAGTGATGTGCTCG	4100-4118	1964
	R	ATATAGGTAATGAGAGCAGATGTG	6040-6063	
**D**	F	AAATAATATGCGTGCCACCT	5434-5453	1661
	R	GAACGCAGAGTAGAAAAGAATA	7073-7094	
**E**	F	CAAGAACACCTGAATTTTATCCCG	6686-6909	2260
	R	TTAGATGCCTTTGGACCTGTTTTA	8922-8945	
**F**	F	TGGTTTCACTCAAAATGGTCC	8876-8896	1223
	R	ATCCCTTCTGCCATTACCTG	10079-10098	
**G**	F	ACCCTTGAGTACCTAAGAGATGA	9965-9987	2116
	R	TGTCCCCATAAGCCCAGAT	12062-12080	
**H**	F	AATCCTGATACCATAGAACTTGT	11801-11823	2032
	R	GTCCGAAATGTCGCTGTG	13815-13832	
**I**	F	CTAGGAAGAGCCTTAATTTGAT	13350-13371	1838
	R	ACAAAGATTTGGTGAATGACA	15167-15187	

a: forward primer; b: reverse primer.

### Amplification of the GC-rich 5'-NCR of the NP gene by RT-PCR

In order to obtain the exact sequence of 5' NCR of NP gene, a 750 bp GC-rich PCR product was amplified with specific primers Pgc Forward (5'- TGG ACC ATC TCA AGA TAA CGA CAC CGA CTG -3') and Pgc Reverse (5'-GTC TTG AGT TGT GTG TCG CCG GCT TCG TC-3'). PCR reaction was carried out by DNA Polymerase with GC-rich buffer (Takara Biotechnology, Dalian, China) according to the manufacturer's instructions. The annealing temperature was 62°C.

### Amplification of the 3'- and 5'-ends of the viral genome

3'- and 5'- ends of the viral genomes were obtained by rapid amplification of cDNA end (RACE) as reported elsewhere [40]. 3'-RACE was carried out by genomic RNA ligation with 5' end phosphated adaptor CL+ (5'-CGC CAG GGT TTT CCC AGT CAC GAC-3'). Our protocols are as follows: viral RNA, 25 pmol of CL+, 2.5 μl of 10×T4 RNA ligase buffer, 1 μl of 10 mM ATP, 2.5 μl of 1 mg/ml BSA, 20 U of RNasin (Takara Biotechnology, Dalian, China), 30 U of T4 RNA ligase (Fermentas, Shenzhen, China), and RNase-free water to a final volume of 25 μl were mixed and incubated at 4°C for 18 h. Then the enzyme was denatured at 75°C for 15 min, and the ligated products were precipitated by ethanol and dissolved in 20 μl RNase-free water. A 5 μl aliquot was taken out to make cDNA with anti-adaptor CL-, which was complementary to adaptor primer CL+. The reaction was conducted with Mo-MLV Reverse Transcriptase (Promega, Madison, WI) according to the manufacturer's instructions. The PCR was carried out with CL- as the forward primer, while the reverse primer 3TSR (5'-GAG AGA TAT GAG AGC ACC TTG TCT GAG T-3') was specific for NP gene of the virus.

For the 5'-RACE, the first strand cDNA was synthesized by Mo-MLV Reverse Transcriptase (Promega, Madison, WI) using a specific primer 5TLF, 5'-GTC CAT TCT GTG CAG AGA GTT TAG TGA G-3', which was located from 14,502 nt to 14,527 nt in the viral genome (mentioned in the direction of 3' end to 5' end of genomic RNA). The reaction mixture was incubated at 42°C for 60 min, and then the cDNA was treated with an equal volume of 0.6 N NaOH for 20 min at 60°C to hydrolyze the mRNA and denature the first-strand cDNA. After purification by using PCR purification kit (Axygen, Union City, CA), the cDNA was ligated with adaptor CL+ by T4 RNA ligase according to procedures as described in 3' RACE. The resulting adaptor-ligated cDNA was amplified using primer 5TLF and anti-adaptor primer CL-. Hemi-nested PCR reaction was then conducted using the primer CL- and specific primer 5TSF, 5'-CAA TAC TGG GTC TCA GAG TCA AAA ATC-3', which was located from 14724 nt to 14751 nt in the viral genome (mentioned in the direction of 3' to 5' of genomic RNA), and then 1 μl of 1:100 diluted primary PCR product was used as template.

### Cloning and sequencing of the amplified products

RT-PCR products of overlapping fragments covering entire genome and GC-rich 5' NCR of NP gene and RT-PCR products by RACE were extracted from agarose gel, ligated into the TA cloning system (Promega, Madison, WI), then transferred into *E. coli *DH5α strain. At least four clones of each segment were sequenced in both directions using the ABI-3700-based (Applied Biosystems Inc.) fluorescent cycle sequencing technology by Sangon Biotechnology (Shanghai, China), and then the correct sequences were determined.

### Sequence analysis

Prediction of amino acid sequences, aligment of sequences and phylogenetic analysis were conducted using the MegAlign program (Windows 32, MegAlign 4.00) in the Lasergene package (DNASTAR Inc. Madison, WI 53715, USA). The sequences of overlapping DNA fragments were aligned and compiled into complete genome. Phylogenetic analysis was performed by using the Lasergene software package and MEGA version 4 (Tamura, Dudley, Nei, and Kumar 2007). Data and accession numbers of complete genome sequences of NDV strains in this study were presented in additional file 1 Table S1. Additional F gene sequences used in Figure 2 were taken from the EMBL/GenBank, the origins of which were described previously [8].

## Abbreviations

APMV: avian paramyxovirus; CEFs: chicken embryo fibroblasts; NCR: Non-coding region; ND: Newcastle disease; NDV: Newcastle disease virus; RACE: rapid amplification of cDNA end; RT-PCR: reverse-transcription polymerase chain reaction.

## Competing interests

The authors declare that they have no competing interests.

## Authors' contributions

XQ, QS, SW and CM contributed for RT-PCR, sequence analysis and generation of phylogenetic tree. Shuang Wu isolated and collected viruses used in this study. LD performed the virus propagation in eggs. XQ and XL drafted the manuscript. XL, SH and YW coordinated overall planning and designed this study. All authors have read and approved the final manuscript.

## Supplementary Material

Additional file 1 **Table S1: Background information of NDV strains with complete genome sequences used in this study**. The genotyping, accession number and references of those NDV strains are shown. Click here for file

## References

[B1] Alexander DJ Newcastle disease, other avian paramyxoviruses, and pneumovirus infections Diseases of Poultry 2003 11 Ames, Iowa: Iowa State University Press 63 69

[B2] Shimazu Y Takao SI Irie T Kiyotani K Yoshida T Sakaguchi T Contribution of the leader sequence to homologous viral interference among Sendai virus strains Virology 2008 372 1 64 71 10.1016/j.virol.2007.10.026 18035388

[B3] Aldous EW Alexander DJ Detection and differentiation of Newcastle disease virus (avian paramyxovirus type 1) Avian Pathol 2001 30 2 117 128 10.1080/03079450120044515 19184885

[B4] de Leeuw O Peeters B Complete nucleotide sequence of Newcastle disease virus: evidence for the existence of a new genus within the subfamily Paramyxovirinae J Gen Virol 1999 80 Pt 1 131 136 9934695 10.1099/0022-1317-80-1-131

[B5] Mayo MA A summary of taxonomic changes recently approved by ICTV Arch Virol 2002 147 8 1655 1663 10.1007/s007050200039 12181683

[B6] Krishnamurthy S Samal SK Nucleotide sequences of the trailer, nucleocapsid protein gene and intergenic regions of Newcastle disease virus strain Beaudette C and completion of the entire genome sequence J Gen Virol 1998 79 Pt 10 2419 2424 9780047 10.1099/0022-1317-79-10-2419

[B7] Huang Y Wan HQ Liu HQ Wu YT Liu XF Genomic sequence of an isolate of Newcastle disease virus isolated from an outbreak in geese: a novel six nucleotide insertion in the non-coding region of the nucleoprotein gene. Brief Report Arch Virol 2004 149 7 1445 1457 10.1007/s00705-004-0297-8 15221544

[B8] Czeglédi A Ujvári D Somogyi E Wehmann E Werner O Lomniczi B Third genome size category of avian paramyxovirus serotype 1 (Newcastle disease virus) and evolutionary implications Virus Res 2006 120 1-2 36 48 16766077 10.1016/j.virusres.2005.11.009

[B9] Millar NS Chambers P Emmerson PT Nucleotide sequence of the fusion and haemagglutinin-neuraminidase glycoprotein genes of Newcastle disease virus, strain Ulster: molecular basis for variations in pathogenicity between strains J Gen Virol 1988 69 Pt 3 613 620 10.1099/0022-1317-69-3-613 3351479

[B10] Samson AC Levesley I Russell PH The 36 K polypeptide synthesized in Newcastle disease virus-infected cells possesses properties predicted for the hypothesized 'V' protein J Gen Virol 1991 72 Pt 7 1709 1713 10.1099/0022-1317-72-7-1709 1856697

[B11] Steward M Vipond IB Millar NS Emmerson PT RNA editing in Newcastle disease virus J Gen Virol 1993 74 Pt 12 2539 2547 10.1099/0022-1317-74-12-2539 8277263

[B12] Gould AR Hansson E Selleck K Kattenbelt JA Mackenzie M Della-Porta AJ Newcastle disease virus fusion and haemagglutinin-neuraminidase gene motifs as markers for viral lineage Avian Pathol 2003 32 4 361 373 10.1080/0307945031000121112 17585460

[B13] Alexander DJ Campbell G Manvell RJ Collins MS Parsons G McNulty MS Characterisation of an antigenically unusual virus responsible for two outbreaks of Newcastle disease in the Republic of Ireland in 1990 Vet Rec 1992 130 4 65 68 10.1136/vr.130.4.65 1532467

[B14] Mia Kim L King DJ Suarez DL Wong CW Afonso CL Characterization of class I Newcastle disease virus isolates from Hong Kong live bird markets and detection using real-time reverse transcription-PCR J Clin Microbiol 2007 45 4 1310 1314 10.1128/JCM.02594-06 17287322 PMC1865838

[B15] Bjarnadottir H Gudmundsson B Gudnason J Jonsson JJ Encapsidation determinants located downstream of the major splice donor in the maedi-visna virus leader region J Virol 2006 80 23 11743 11755 10.1128/JVI.01284-06 16971429 PMC1642619

[B16] Liu X Wang X Wu S Hu S Peng Y Xue F Surveillance for avirulent Newcastle disease viruses in domestic ducks (Anas platyrhynchos and Cairina moschata) at live bird markets in Eastern China and characterization of the viruses isolated Avian Pathol 2009 38 5 377 391 10.1080/03079450903183637 19937525

[B17] Seal BS Wise MG Pedersen JC Senne DA Alvarez R Scott MS King DJ Yu Q Kapczynski DR Genomic sequences of low-virulence avian paramyxovirus-1 (Newcastle disease virus) isolates obtained from live-bird markets in North America not related to commonly utilized commercial vaccine strains Vet Microbiol 2005 106 1-2 7 16 10.1016/j.vetmic.2004.11.013 15737469

[B18] Ujvári D Wehmann E Herczeg J Lomniczi B Identification and subgrouping of pigeon type Newcastle disease virus strains by restriction enzyme cleavage site analysis J Virol Methods 2006 131 2 115 121 16146659 10.1016/j.jviromet.2005.07.012

[B19] Lomniczi B Wehmann E Herczeg J Ballagi-Pordany A Kaleta EF Werner O Meulemans G Jorgensen PH Mante AP Gielkens AL Newcastle disease outbreaks in recent years in western Europe were caused by an old (VI) and a novel genotype (VII) Arch Virol 1998 143 1 49 64 10.1007/s007050050267 9505965

[B20] Herczeg J Wehmann E Bragg RR Travassos Dias PM Hadjiev G Werner O Lomniczi B Two novel genetic groups (VIIb and VIII) responsible for recent Newcastle disease outbreaks in Southern Africa, one (VIIb) of which reached Southern Europe Arch Virol 1999 144 11 2087 2099 10.1007/s007050050624 10603164

[B21] Liu XF Wan HQ Ni XX Wu YT Liu WB Pathotypical and genotypical characterization of strains of Newcastle disease virus isolated from outbreaks in chicken and goose flocks in some regions of China during 1985-2001 Arch Virol 2003 148 7 1387 1403 12827467 10.1007/s00705-003-0014-z

[B22] Liang Y Ma WT A fowl disease caused by a filterable virus Agricult Bull (in Chinese) 1946 12 14 16

[B23] Liang R Cao DJ Li JQ Chen J Guo X Zhuang FF Duan MX Newcastle disease outbreaks in western China were caused by the genotypes VIIa and VIII Vet Microbiol 2002 87 3 193 203 10.1016/S0378-1135(02)00050-0 12052330

[B24] Miller PJ Decanini EL Afonso CL Newcastle disease: evolution of genotypes and the related diagnostic challenges Infect Genet Evol 10 1 26 35 10.1016/j.meegid.2009.09.012 19800028

[B25] Yu L Wang Z Jiang Y Chang L Kwang J Characterization of newly emerging Newcastle disease virus isolates from the People's Republic of China and Taiwan J Clin Microbiol 2001 39 10 3512 3519 10.1128/JCM.39.10.3512-3519.2001 11574565 PMC88381

[B26] Qin ZM Tan LT Xu HY Ma BC Wang YL Yuan XY Liu WJ Pathotypical characterization and molecular epidemiology of Newcastle disease virus isolates from different hosts in China from 1996 to 2005 J Clin Microbiol 2008 46 2 601 611 10.1128/JCM.01356-07 18077643 PMC2238121

[B27] Ronin C Bouchilloux S Granier C van Rietschoten J Enzymatic N-glycosylation of synthetic Asn--X--Thr containing peptides FEBS Lett 1978 96 1 179 182 10.1016/0014-5793(78)81089-8 729783

[B28] Hart GW Brew K Grant GA Bradshaw RA Lennarz WJ Primary structural requirements for the enzymatic formation of the N-glycosidic bond in glycoproteins. Studies with natural and synthetic peptides J Biol Chem 1979 254 19 9747 9753 489565

[B29] Connaris H Takimoto T Russell R Crennell S Moustafa I Portner A Taylor G Probing the sialic acid binding site of the hemagglutinin-neuraminidase of Newcastle disease virus: identification of key amino acids involved in cell binding, catalysis, and fusion J Virol 2002 76 4 1816 1824 10.1128/JVI.76.4.1816-1824.2002 11799177 PMC135884

[B30] Takimoto T Taylor GL Crennell SJ Scroggs RA Portner A Crystallization of Newcastle disease virus hemagglutinin-neuraminidase glycoprotein Virology 2000 270 1 208 214 10.1006/viro.2000.0263 10772993

[B31] Iorio RM Field GM Sauvron JM Mirza AM Deng R Mahon PJ Langedijk JP Structural and functional relationship between the receptor recognition and neuraminidase activities of the Newcastle disease virus hemagglutinin-neuraminidase protein: receptor recognition is dependent on neuraminidase activity J Virol 2001 75 4 1918 1927 10.1128/JVI.75.4.1918-1927.2001 11160691 PMC115138

[B32] Sakaguchi T Toyoda T Gotoh B Inocencio NM Kuma K Miyata T Nagai Y Newcastle disease virus evolution. I. Multiple lineages defined by sequence variability of the hemagglutinin-neuraminidase gene Virology 1989 169 2 260 272 10.1016/0042-6822(89)90151-7 2705297

[B33] Locke DP Sellers HS Crawford JM Schultz-Cherry S King DJ Meinersmann RJ Seal BS Newcastle disease virus phosphoprotein gene analysis and transcriptional editing in avian cells Virus Res 2000 69 1 55 68 10.1016/S0168-1702(00)00175-1 10989186

[B34] Seal BS King DJ Locke DP Senne DA Jackwood MW Phylogenetic relationships among highly virulent Newcastle disease virus isolates obtained from exotic birds and poultry from 1989 to 1996 J Clin Microbiol 1998 36 4 1141 1145 9542957 10.1128/jcm.36.4.1141-1145.1998 PMC104709

[B35] Seal BS King DJ Meinersmann RJ Molecular evolution of the Newcastle disease virus matrix protein gene and phylogenetic relationships among the paramyxoviridae Virus Res 2000 66 1 1 11 10.1016/S0168-1702(99)00119-7 10653913

[B36] Wise MG Sellers HS Alvarez R Seal BS RNA-dependent RNA polymerase gene analysis of worldwide Newcastle disease virus isolates representing different virulence types and their phylogenetic relationship with other members of the paramyxoviridae Virus Res 2004 104 1 71 80 10.1016/j.virusres.2004.01.034 15177894

[B37] Taniura N Saito M Okuwa T Saito K Ohara Y Different subcellular localization of Theiler's murine encephalomyelitis virus leader proteins of GDVII and DA strains in BHK-21 cells J Virol 2009 83 13 6624 6630 10.1128/JVI.02385-08 19386716 PMC2698518

[B38] Plumet S Herschke F Bourhis JM Valentin H Longhi S Gerlier D Cytosolic 5'-triphosphate ended viral leader transcript of measles virus as activator of the RIG I-mediated interferon response PLoS One 2007 2 3 e279 10.1371/journal.pone.0000279 17356690 PMC1804102

[B39] Qiu X Sun Q Yao C Dong L Wu Y Hu S Liu X [Full-length genome analysis of two genotype III velogenic Newcastle diseases virus strains reveals their close relationship with vaccine Mukteswar] Wei Sheng Wu Xue Bao 2009 49 3 302 308 19623952

[B40] Ricour C Borghese F Sorgeloos F Hato SV van Kuppeveld FJ Michiels T Random mutagenesis defines a domain of Theiler's virus leader protein that is essential for antagonism of nucleocytoplasmic trafficking and cytokine gene expression J Virol 2009 83 21 11223 11232 10.1128/JVI.00829-09 19710133 PMC2772802

