# Supplementary material for: Entire genome sequence analysis of genotype IX Newcastle disease viruses reveals their early-genotype phylogenetic position and recent-genotype genome size
Source: Virol J. 2011 Mar 14;8:117. doi: 10.1186/1743-422X-8-117 (PMC3063233; doi:10.1186/1743-422X-8-117)
Supplement: Additional file 1 — Table S1: Background information of NDV strains with complete genome sequences used in this study. The genotyping, accession number and references of those NDV strains are shown. [file 1743-422X-8-117-S1.DOC]

**Table S1** Background information of NDV strains with complete genome sequences used in this study

| **Division** | **Genotype** | **Virus strain** | **Accession number** | **References** |
| --- | --- | --- | --- | --- |
| Class I |  | DE-R49/99 | DQ097393 | Czeglédi *et al.* |
| Class II | I | PHY-LMV42/66 | DQ097394 |
|  |  | I-2 | AY935499 | Kattenbelt, Meers, and Gould |
|  |  | AU-1252/98 | AY935493 | Kattenbelt, Stevens, and Gould |
|  |  | AU-1334/02 | AY935490 |
|  |  | CN/Ulster/67 | AY562991 | Wise *et al.* |
|  | II | B1/46 | NC002617 | Sellers and Seal (2006), unpublished results |
|  |  | LaSota/46 | AF077761 | de Leeuw and Peeters |
|  |  | AQI-ND026 | DQ060053 | Wang *et al.* (2005), unpublished results |
|  |  | VG/GA | EU289028 | [Perozo](../../../../E:%5Csites%5Centrez%3FDb=pubmed&Cmd=Search&Term=%22Perozo%20F%22%5BAuthor%5D&itool=EntrezSystem2.PEntrez.Pubmed.Pubmed_ResultsPanel.Pubmed_DiscoveryPanel.Pubmed_RVAbstractPlus), Villegas, and Afonso |
|  | III | Mukteswar | [EF201805](../../../../E:%5Centrez%5Cviewer.fcgi%3Fdb=nucleotide&val=124014023) | Li and Liu (2007), unpublished results |
|  |  | JS/7/05/Ch | FJ430159 | Qiu *et al.* (2008), unpublished results |
|  |  | JS/9/05/Go | FJ430160 |
|  | IV | Herts/33 | AY741404 | De Leeuw *et al.* |
|  |  | Italien | EU293914.1 | Wei *et al.* |
|  | V | US(FL)/Largo/71 | AY562990 | Wise *et al.* |
|  |  | US(CA)/211472/02 | AY562987 |
|  |  | US(FL)/44083/93 | AY562986 |
|  | VI | US(CA)/1083Fontana/72 | AY562988 |
|  |  | dove/Italy/2736/00 | AY562989 | Wise *et al.* (2004), unpublished results |
|  |  | IT-227/82 | AJ880277 | Ujvári *et al.* |
|  | VII | CN/ZJ-1/00 | AF431744 | Huang *et al.* |
|  |  | NA-1 | DQ659677 | Xu *et al.* |
|  |  | CN/Guangxi7/02 | DQ485229 | Xie *et al.* (2006), unpublished results |
|  |  | CN/Guangxi9/02 | DQ485230 |
|  |  | CN/Guangxi11/02 | DQ485231 |

Czeglédi, A., Ujvári, D., Somogyi, E., Wehmann, E., Werner, O., and Lomniczi, B., 2006. Third genome size category of avian paramyxovirus serotype 1 (Newcastle disease virus) and evolutionary implications. *Virus Res* 120**,** 36-48.

de Leeuw, O., and Peeters, B., 1999. Complete nucleotide sequence of Newcastle disease virus: evidence for the existence of a new genus within the subfamily Paramyxovirinae. *J Gen Virol* 80 ( Pt 1)**,** 131-136.

de Leeuw, O. S., Koch, G., Hartog, L., Ravenshorst, N., and Peeters, B. P., 2005. Virulence of Newcastle disease virus is determined by the cleavage site of the fusion protein and by both the stem region and globular head of the haemagglutinin-neuraminidase protein. *J Gen Virol* 86**,** 1759-1769.

Huang, Y., Wan, H. Q., Liu, H. Q., Wu, Y. T., and Liu, X. F., 2004. Genomic sequence of an isolate of Newcastle disease virus isolated from an outbreak in geese: a novel six nucleotide insertion in the non-coding region of the nucleoprotein gene. Brief Report. *Arch Virol* 149**,** 1445-1457.

Kattenbelt, J. A., Meers, J., and Gould, A. R., 2006. Genome sequence of the thermostable Newcastle disease virus (strain I-2) reveals a possible phenotypic locus. *Vet Microbiol* 114**,** 134-141.

Kattenbelt, J. A., Stevens, M. P., and Gould, A. R., 2006. Sequence variation in the Newcastle disease virus genome. *Virus Res* 116**,** 168-184.

Perozo, F., Villegas, P., and Afonso, C. L., 2008. Genomic comparison of the complete coding and intergenic regions of the VG/GA Newcastle disease virus and its respirotropic clone 5. *Virus Genes* 37**,** 161-167.

Ujvari, D., Wehmann, E., Herczeg, J., and Lomniczi, B., 2006. Identification and subgrouping of pigeon type Newcastle disease virus strains by restriction enzyme cleavage site analysis. *J Virol Methods* 131**,** 115-121.

Wei, D., Yang, B., Li, Y. L., Xue, C. F., Chen, Z. N., and Bian, H., 2008. Characterization of the genome sequence of an oncolytic Newcastle disease virus strain Italien. *Virus Res* 135**,** 312-319.

Wise, M. G., Sellers, H. S., Alvarez, R., and Seal, B. S., 2004. RNA-dependent RNA polymerase gene analysis of worldwide Newcastle disease virus isolates representing different virulence types and their phylogenetic relationship with other members of the paramyxoviridae. *Virus Res* 104**,** 71-80.

Xu, M., Chang, S., Ding, Z., Gao, H. W., Wan, J. Y., Liu, W. S., Liu, L. N., Gao, Y., and Xu, J., 2008. Genomic analysis of Newcastle disease virus strain NA-1 isolated from geese in China. *Arch Virol* 153**,** 1281-1289.
